# Clinical factors associated with recurrent pregnancy loss status in women with polyendocrine metabolic ovarian syndrome

**DOI:** 10.3389/fendo.2026.1897833

**Published:** 2026-09-10

**Authors:** Cong Sheng, Hui-Fen Zhang, Xin Zhang, Tao Fan, Li-Qin Cheng

**Affiliations:** 1 Department of Obstetrics, The Eighth Affiliated Hospital of Sun Yat-sen University, Shenzhen, Guangdong, China; 2 Department of Gynecology, The Eighth Affiliated Hospital of Sun Yat-sen University, Shenzhen, Guangdong, China; 3 Department of Gynecology, Futian Maternal and Child Health Hospital of Shenzhen, Shenzhen, Guangdong, China

**Keywords:** insulin resistance, polyendocrine metabolic ovarian syndrome, prediction model, recurrent pregnancy loss, risk factors

## Abstract

**Background:**

Women with polyendocrine metabolic ovarian syndrome (PMOS) may have an increased susceptibility to recurrent pregnancy loss (RPL), but the clinical factors associated with RPL status remain incompletely characterized. This study aimed to identify factors associated with RPL status and to develop an internally validated discriminative model in women with PMOS.

**Methods:**

This retrospective, single-center study included 235 women with PMOS and at least one documented pregnancy between January 2022 and December 2025. Participants were classified into the RPL group (n = 70) or non-RPL group (n = 165). Univariable and multivariable logistic regression analyses were performed. Model discrimination, calibration, and internal validity were evaluated using receiver operating characteristic analysis and bootstrap resampling.

**Results:**

Older age (adjusted odds ratio [OR] = 1.06, 95% confidence interval [CI]: 1.01–1.12), higher body mass index (OR = 1.10, 95% CI: 1.02–1.19), infertility history (OR = 2.01, 95% CI: 1.11–3.64), higher luteinizing hormone/follicle-stimulating hormone ratio (OR = 1.77, 95% CI: 1.18–2.67), higher homeostasis model assessment of insulin resistance (OR = 1.41, 95% CI: 1.15–1.73), and higher D-dimer concentration (OR = 6.06, 95% CI: 1.92–19.15) were independently associated with higher odds of RPL status. Previous live birth was inversely associated with RPL status (OR = 0.35, 95% CI: 0.18–0.68). The model achieved an apparent area under the curve of 0.846 (95% CI: 0.791–0.901) and a bootstrap-corrected value of 0.831.

**Conclusions:**

Reproductive, endocrine, metabolic, and coagulation-related factors were associated with RPL status in women with PMOS. The resulting model showed good internal discrimination but requires external prospective validation before clinical application.

## 1 Introduction

Polyendocrine metabolic ovarian syndrome (PMOS), formerly known as polycystic ovary syndrome (PCOS), is a common reproductive and endocrine disorder characterized by ovulatory dysfunction, hyperandrogenism, and polycystic ovarian morphology, with substantial heterogeneity in clinical presentation and reproductive outcomes. The term PMOS was adopted following a 2026 global consensus to better reflect the broader endocrine and metabolic features of the condition (1, 2). Beyond menstrual irregularity and subfertility, PMOS is associated with adverse pregnancy outcomes, including miscarriage and recurrent pregnancy loss (RPL) (3). Emerging evidence suggests that these outcomes may not be explained by anovulation alone but may also involve impaired endometrial receptivity, trophoblast dysfunction, metabolic dysregulation, and abnormal placental development (4–6). RPL is a clinically important and etiologically heterogeneous reproductive disorder. Among women with PMOS, susceptibility to pregnancy loss may reflect the combined effects of endocrine, metabolic, and reproductive abnormalities rather than a single pathogenic mechanism (7, 8). Hyperandrogenism has been associated with defective decidualization and impaired embryo–maternal interactions (9). Mechanistic studies have further suggested that androgen excess may compromise trophoblast viability through ferroptosis-related pathways and impair villous development through mitochondrial dysfunction, providing a biologically plausible explanation for pregnancy failure in some PMOS phenotypes (10, 11).

Insulin resistance is another central pathophysiological feature of PMOS and has been associated with impaired fertility, reduced endometrial receptivity, poorer assisted reproductive outcomes, and lower live birth rates. Obesity may further exacerbate insulin resistance, hyperandrogenemia, chronic low-grade inflammation, and dyslipidemia, thereby contributing to an unfavorable reproductive profile (12, 13). Women with both PMOS and obesity often exhibit more pronounced metabolic and reproductive abnormalities, including prolonged time to conception, reduced fertility, and a greater risk of miscarriage. These observations support the clinical relevance of metabolic assessment in women with PMOS and a history of pregnancy loss (12, 14). Endometrial and placental abnormalities may also contribute to reproductive failure in this population (15). Altered extracellular matrix remodeling, disrupted immune–endometrial interactions, and phenotype-related placental changes have been reported, supporting a potential role for impaired implantation and placental development (16). Differences in miscarriage rates across assisted reproductive approaches may further reflect underlying biological heterogeneity among women with PMOS, which may influence pregnancy maintenance even after conception has occurred (17, 18).

Previous studies have linked pregnancy loss in women with PMOS primarily to reproductive, endocrine, and metabolic abnormalities, and existing models in women with this condition have largely focused on early pregnancy loss using hormonal and metabolic variables (19, 20). However, evidence regarding coagulation-related markers in PMOS-associated RPL remains limited and inconsistent. In particular, whether D-dimer is associated with RPL status after consideration of reproductive history, gonadotropic indices, and insulin resistance remains insufficiently characterized (21, 22). To address this gap, this study aimed to investigate clinical factors associated with RPL status in women with PMOS, with particular attention to D-dimer, and to develop an internally validated multivariable model for distinguishing women with and without a documented history of RPL.

## 2 Methods

### 2.1 Study design

This retrospective, single-center study included women with PMOS who underwent evaluation and treatment at our institution between January 2022 and December 2025. Eligible participants were women of reproductive age with a confirmed diagnosis of PMOS, at least one documented pregnancy, and sufficient medical records to determine previous pregnancy outcomes and RPL status. The patient was the unit of analysis. Participants were excluded if the available medical records documented a recognized alternative explanation for pregnancy loss, including uterine anatomical abnormalities, chromosomal abnormalities in either partner, autoimmune disease, antiphospholipid syndrome, overt thyroid dysfunction, isolated thyroid autoantibody positivity with normal thyroid function, uncontrolled diabetes mellitus, hyperprolactinemia, inherited or acquired thrombophilia, severe hepatic or renal dysfunction, or another major endocrine or systemic disorder with a potential effect on pregnancy outcomes. Participants without a definite pregnancy history or with insufficient key data to determine RPL status or complete the primary analysis were also excluded. Uterine anatomy was evaluated by transvaginal ultrasonography in all included participants. Three-dimensional ultrasonography, hysteroscopy, hysterosalpingography, or pelvic magnetic resonance imaging was performed when clinically indicated. Maternal and partner chromosomal abnormalities were assessed by peripheral blood karyotyping when performed. When available, screening for antiphospholipid syndrome comprised lupus anticoagulant, anticardiolipin antibodies, and anti-β2-glycoprotein I antibodies. Thyroid evaluation included serum thyroid-stimulating hormone and free thyroxine, with thyroid peroxidase antibody and thyroglobulin antibody results recorded when available. Autoimmune disease and thrombophilia were assessed using documented medical history, available laboratory findings, and clinically indicated antibody or institutional thrombophilia testing. Because these additional etiological assessments were not uniformly performed in this retrospective cohort, the absence of a documented assessment was not interpreted as a normal or negative result. The study was approved by the ethics committee of our hospital and conducted in accordance with the Declaration of Helsinki. All data were anonymized before analysis to protect participant confidentiality.

### 2.2 Diagnostic criteria and grouping

A documented diagnosis of PMOS was retrospectively confirmed in accordance with the Rotterdam criteria, as recommended by the 2023 International Evidence-based Guideline for the Assessment and Management of Polycystic Ovary Syndrome. PMOS was defined by the presence of at least two of the following three features after exclusion of other relevant etiologies: (1) oligo-ovulation or anovulation, (2) clinical and/or biochemical hyperandrogenism, and (3) polycystic ovarian morphology on ultrasonography. Oligo-ovulation or anovulation was identified from documented menstrual irregularity, including oligomenorrhea or amenorrhea. Clinical hyperandrogenism was identified from documented manifestations such as hirsutism, acne, or androgenic alopecia, whereas biochemical hyperandrogenism was based on elevated circulating androgen concentrations. Polycystic ovarian morphology was determined from available pelvic ultrasonography reports according to accepted sonographic criteria. Disorders that may mimic PMOS, including congenital adrenal hyperplasia, androgen-secreting tumors, Cushing syndrome, thyroid dysfunction, and hyperprolactinemia, were excluded on the basis of the available clinical and laboratory records (23).

RPL was defined in accordance with the European Society of Human Reproduction and Embryology guideline as the loss of two or more pregnancies. In the present study, a qualifying pregnancy loss was defined as a documented spontaneous pregnancy loss occurring before 24 completed gestational weeks. Both consecutive and non-consecutive pregnancy losses were included. Pregnancy-loss episodes were retrospectively ascertained from outpatient and inpatient records, ultrasonography reports, laboratory results, and available follow-up documentation.Pregnancy losses confirmed by ultrasonography were classified as clinical pregnancy losses. Pregnancy losses documented solely by serial serum human chorionic gonadotropin measurements without ultrasonographic confirmation were classified as biochemical pregnancy losses and were included in the primary RPL definition. Ectopic pregnancy, molar pregnancy, and elective termination of pregnancy were not counted as qualifying pregnancy losses (24). Consecutive RPL was defined as at least two qualifying pregnancy losses occurring in successive pregnancies without an intervening ongoing pregnancy or live birth. Non-consecutive RPL was defined as at least two qualifying pregnancy losses separated by one or more pregnancies that did not end in a qualifying loss. Primary RPL referred to RPL occurring in women with no previous live birth, whereas secondary RPL referred to RPL occurring after at least one previous live birth. Based on the retrospectively ascertained pregnancy history, participants with at least two qualifying pregnancy losses were classified into the RPL group, whereas those with at least one documented pregnancy who did not meet the RPL criteria were classified into the non-RPL group.

### 2.3 Data collection

Demographic, clinical, reproductive, ultrasonographic, and laboratory data were retrospectively extracted from the electronic medical record system, inpatient and outpatient records, laboratory databases, imaging reports, and available follow-up documentation.

Demographic and general clinical variables included age, body weight, height, body mass index (BMI), marital status, smoking history, and alcohol consumption history. BMI was calculated as body weight in kilograms divided by height in meters squared. Smoking history was defined as documented current or previous tobacco use, and alcohol consumption history was defined as documented current or previous regular alcohol intake.

Reproductive variables included menstrual pattern, gravidity, number of documented qualifying pregnancy losses, nulliparity, history of infertility, duration of infertility, use of assisted reproductive technology (ART), and history of live birth. Menstrual pattern was categorized as regular menstruation, oligomenorrhea, or amenorrhea/marked irregularity according to the documented clinical history. Gravidity was defined as the total number of documented pregnancies, irrespective of pregnancy outcome. The number of documented qualifying pregnancy losses included both ultrasonographically confirmed clinical pregnancy losses and biochemical pregnancy losses meeting the study definition. ART use was defined as documented previous use of an assisted reproductive procedure. An ART-associated pregnancy loss was defined as a qualifying loss following a pregnancy achieved through ART.

A history of live birth was defined as at least one documented previous delivery of a live infant. For the classification of primary and secondary RPL, only live births occurring before fulfillment of the RPL criteria were considered. Duration of infertility was defined as the documented interval from the initiation of regular unprotected intercourse to the diagnosis of infertility and was recorded only for women with a documented history of infertility.

For each documented pregnancy-loss episode, the available information included gestational age at loss, date and sequence of the loss, ultrasonographic findings, serial serum human chorionic gonadotropin results, mode of conception, and the occurrence of an intervening pregnancy or live birth. Ovarian ultrasonographic variables included antral follicle count, ovarian volume, and the presence of polycystic ovarian morphology.

Endocrine variables included luteinizing hormone (LH), follicle-stimulating hormone (FSH), LH/FSH ratio, total testosterone, estradiol, and prolactin. The LH/FSH ratio was calculated by dividing the serum LH concentration by the serum FSH concentration. Metabolic variables included fasting plasma glucose, fasting insulin, homeostasis model assessment of insulin resistance (HOMA-IR), total cholesterol, triglycerides, high-density lipoprotein cholesterol, and low-density lipoprotein cholesterol. HOMA-IR was calculated as follows: HOMA-IR= {fasting insulin (μU/mL)×fasting plasma glucose (mmol/L)}/22.5. Thyroid-related variables included thyroid-stimulating hormone, free triiodothyronine, and free thyroxine. Thyroid peroxidase antibody and thyroglobulin antibody results were additionally recorded when available to characterize the completeness of thyroid autoimmunity assessment. Coagulation-related variables included prothrombin time, activated partial thromboplastin time, fibrinogen, and D-dimer. Laboratory results were retrieved from the hospital laboratory database and had been measured according to standard institutional procedures. For participants with repeated measurements, the value obtained closest to the index clinical evaluation was selected. The index clinical evaluation was defined as the earliest eligible encounter during the study period at which PMOS status, pregnancy history, and the required clinical variables could be ascertained.

Because D-dimer concentrations may vary according to pregnancy status and concurrent clinical conditions, the context of each selected D-dimer measurement was retrospectively reviewed. Extracted information included pregnancy status at measurement, the interval from the most recent qualifying pregnancy loss, measurement during the acute pregnancy-loss phase, uterine evacuation or surgery within the preceding 30 days, ovarian stimulation, oocyte retrieval within the preceding 7 days, embryo transfer within the preceding 14 days, current anticoagulant or antiplatelet therapy, active infection or inflammatory disease, and non-obstetric surgery within the preceding 30 days. A stable-baseline D-dimer measurement was defined as one obtained in a nonpregnant state, outside the acute phase of pregnancy loss, and in the absence of recent uterine evacuation, surgery, ovarian stimulation, oocyte retrieval, embryo transfer, active infection or inflammation, or current anticoagulant therapy.

### 2.4 Statistical analysis

All statistical analyses were performed using IBM SPSS Statistics, version 29.0 (IBM Corp., Armonk, NY, USA). Continuous variables were summarized as mean ± standard deviation or median with interquartile range, as appropriate, and categorical variables as number and percentage. Between-group comparisons were performed using the independent-samples t test or Mann–Whitney U test for continuous variables and Pearson’s chi-square test or Fisher’s exact test for categorical variables, as appropriate. No between-group test was performed for the number of qualifying pregnancy losses because this variable contributed directly to RPL classification. For logistic regression analyses, RPL status was coded as 1 and non-RPL status as 0, with the non-RPL group serving as the reference category. Univariable logistic regression was used to estimate odds ratios (ORs) and 95% confidence intervals (CIs). The final multivariable logistic regression model included age, BMI, history of infertility, history of live birth, LH/FSH ratio, HOMA-IR, and D-dimer. Multicollinearity was assessed using tolerance and variance inflation factors. Model discrimination was evaluated using receiver operating characteristic analysis and the area under the curve (AUC), with 95% CIs calculated using the DeLong method. The cutoff maximizing the Youden index was used to calculate sensitivity, specificity, positive and negative predictive values, and overall accuracy. Bootstrap resampling was used for internal validation and estimation of optimism-corrected AUC, calibration intercept, calibration slope, Brier score, and classification performance. Sensitivity analyses reclassified RPL after excluding biochemical pregnancy losses, restricted the analysis to stable-baseline D-dimer measurements, excluded history of live birth or D-dimer from the model, and restricted the cohort to participants with complete documented etiological screening. Duration of infertility was analyzed only among women with infertility, and FT4 was analyzed using available cases. No imputation was performed for the primary model. All tests were two-sided, with P < 0.05 considered statistically significant.

## 3 Results

### 3.1 Study population selection and grouping

A total of 256 women of reproductive age with documented PMOS who were evaluated at our institution between January 2022 and December 2025 were screened for eligibility. Twenty-one were excluded: 6 lacked a definite pregnancy history, 5 lacked key data required to determine RPL status or complete the primary analysis, 4 had uterine anatomical abnormalities, 2 had chromosomal abnormalities in either partner, 2 had overt thyroid dysfunction, 1 had isolated thyroid autoantibody positivity with normal thyroid function, and 1 had another autoimmune or clinically relevant disorder. The final cohort comprised 235 women. Based on retrospectively ascertained pregnancy histories, 70 women with at least two qualifying pregnancy losses were classified into the RPL group, whereas 165 women with at least one documented pregnancy who did not meet the RPL criteria were classified into the non-RPL group.

### 3.2 Demographic, reproductive, and ultrasonographic characteristics

Women in the RPL group were older than those in the non-RPL group (30.8 ± 4.7 vs. 28.9 ± 4.5 years, P = 0.005) and had higher body weight (67.2 ± 10.3 vs. 63.8 ± 9.6 kg, P = 0.020) and BMI (25.8 ± 3.6 vs. 24.3 ± 3.2 kg/m², P = 0.003). Nulliparity (57.1% vs. 43.0%, P = 0.048), a history of infertility (65.7% vs. 47.9%, P = 0.012), and previous ART use (25.7% vs. 12.7%, P = 0.014) were more frequent in the RPL group. In contrast, a history of live birth was less frequent among women with RPL (20.0% vs. 49.1%, P < 0.001).

The RPL group also had a higher mean gravidity (3.2 ± 1.1 vs. 2.4 ± 0.9, P < 0.001). Among women with a documented history of infertility, the duration of infertility was longer in the RPL group than in the non-RPL group (3.8 ± 1.9 vs. 2.9 ± 1.6 years, P = 0.008). Mean ovarian volume was also greater in the RPL group (12.8 ± 3.1 vs. 11.6 ± 2.8 mL, P = 0.006). No statistically significant differences were observed in height, marital status, smoking history, alcohol consumption history, menstrual pattern, antral follicle count, or the prevalence of polycystic ovarian morphology (**Table 1**).

**Table 1:** Demographic, reproductive, and ultrasonographic characteristics.

Variable	RPL group (n = 70)	Non-RPL group (n = 165)	Test statistic	P value
Age, years	30.8 ± 4.7	28.9 ± 4.5	t = 2.87	0.005
Body weight, kg	67.2 ± 10.3	63.8 ± 9.6	t = 2.36	0.020
Height, cm	161.4 ± 5.7	162.1 ± 5.5	t = −0.87	0.386
BMI, kg/m²	25.8 ± 3.6	24.3 ± 3.2	t = 3.02	0.003
Married, n (%)	58 (82.9)	129 (78.2)	χ² = 0.66	0.416
Nulliparity, n (%)	40 (57.1)	71 (43.0)	χ² = 3.93	0.048
Smoking history, n (%)	10 (14.3)	13 (7.9)	χ² = 2.28	0.131
Alcohol consumption history, n (%)	8 (11.4)	12 (7.3)	χ² = 1.09	0.296
History of infertility, n (%)	46 (65.7)	79 (47.9)	χ² = 6.28	0.012
ART use, n (%)	18 (25.7)	21 (12.7)	χ² = 5.99	0.014
Menstrual pattern, n (%)			χ² = 4.23	0.121
└ Regular	8 (11.4)	35 (21.2)		
└ Oligomenorrhea	29 (41.4)	71 (43.0)		
└ Amenorrhea/marked irregularity	33 (47.1)	59 (35.8)		
Gravidity, n	3.2 ± 1.1	2.4 ± 0.9	t = 5.37	<0.001
Number of documented pregnancy losses, n	2.6 ± 0.7	0.5 ± 0.6	—	—
History of live birth, n (%)	14 (20.0)	81 (49.1)	χ² = 17.27	<0.001
Duration of infertility, years	3.8 ± 1.9 (n = 46)	2.9 ± 1.6 (n = 79)	t = 2.70	0.008
Antral follicle count, n	25.1 ± 6.8	23.4 ± 6.1	t = 1.81	0.073
Ovarian volume, mL	12.8 ± 3.1	11.6 ± 2.8	t = 2.79	0.006
Polycystic ovarian morphology, n (%)	62 (88.6)	136 (82.4)	χ² = 1.40	0.237

### 3.3 Endocrine, metabolic, thyroid, and coagulation-related parameters

Women in the RPL group had higher serum LH concentrations (11.8 ± 4.2 vs. 10.2 ± 3.8 IU/L, P = 0.006), LH/FSH ratios (2.03 ± 0.74 vs. 1.67 ± 0.63, P < 0.001), and total testosterone concentrations (2.18 ± 0.71 vs. 1.89 ± 0.62 nmol/L, P = 0.003). FSH, estradiol, and prolactin concentrations did not differ significantly between the groups.

Fasting plasma glucose, fasting insulin, and HOMA-IR were higher in the RPL group than in the non-RPL group (all P ≤ 0.009). The RPL group also had higher total cholesterol, triglyceride, and LDL-C concentrations and lower HDL-C concentrations. Thyroid function parameters, including TSH, FT3, and FT4, were not significantly different between the groups.

Among the coagulation-related parameters, fibrinogen (3.46 ± 0.68 vs. 3.21 ± 0.59 g/L, P = 0.005) and D-dimer (0.41 ± 0.18 vs. 0.32 ± 0.14 mg/L, P < 0.001) were higher in the RPL group. Prothrombin time and activated partial thromboplastin time did not differ significantly (**Table 2**).

**Table 2:** Endocrine, metabolic, thyroid, and coagulation-related parameters.

Variable	RPL group (n = 70)	Non-RPL group (n = 165)	Test statistic	P value
Sex hormone and endocrine parameters				
LH, IU/L	11.8 ± 4.2	10.2 ± 3.8	t = 2.80	0.006
FSH, IU/L	6.1 ± 1.7	6.4 ± 1.8	t = −1.17	0.243
LH/FSH ratio	2.03 ± 0.74	1.67 ± 0.63	t = 3.71	<0.001
Total testosterone, nmol/L	2.18 ± 0.71	1.89 ± 0.62	t = 3.05	0.003
Estradiol, pmol/L	214.6 ± 67.9	226.8 ± 71.4	t = −1.21	0.227
Prolactin, ng/mL	17.9 ± 6.3	16.8 ± 5.7	t = 1.31	0.192
Glucose metabolism and insulin resistance parameters				
Fasting plasma glucose, mmol/L	5.41 ± 0.63	5.19 ± 0.57	t = 2.62	0.009
Fasting insulin, μU/mL	15.8 ± 5.6	12.9 ± 4.8	t = 4.04	<0.001
HOMA-IR	3.82 ± 1.41	2.98 ± 1.19	t = 4.61	<0.001
Lipid profile parameters				
Total cholesterol, mmol/L	5.12 ± 0.86	4.89 ± 0.79	t = 2.00	0.047
Triglycerides, mmol/L	1.83 ± 0.61	1.49 ± 0.54	t = 4.16	<0.001
HDL-C, mmol/L	1.12 ± 0.21	1.21 ± 0.24	t = −2.77	0.006
LDL-C, mmol/L	3.18 ± 0.73	2.97 ± 0.69	t = 2.07	0.039
Thyroid function parameters				
TSH, mIU/L	2.41 ± 0.98	2.16 ± 0.87	t = 1.91	0.058
FT3, pmol/L	4.82 ± 0.51	4.88 ± 0.47	t = −0.86	0.391
FT4, pmol/L	14.3 ± 2.1 (n = 68)	14.6 ± 2.0 (n = 159)	t = −1.00	0.319
Coagulation-related parameters				
Prothrombin time, s	11.7 ± 0.8	11.5 ± 0.7	t = 1.93	0.055
Activated partial thromboplastin time, s	30.8 ± 3.1	31.2 ± 3.0	t = −0.92	0.360
Fibrinogen, g/L	3.46 ± 0.68	3.21 ± 0.59	t = 2.85	0.005
D-dimer, mg/L	0.41 ± 0.18	0.32 ± 0.14	t = 4.10	<0.001

Data are presented as mean ± standard deviation. FT4 was available for 68 women in the RPL group and 159 women in the non-RPL group.

LH, luteinizing hormone; FSH, follicle-stimulating hormone; HOMA-IR, homeostasis model assessment of insulin resistance; HDL-C, high-density lipoprotein cholesterol; LDL-C, low-density lipoprotein cholesterol; TSH, thyroid-stimulating hormone; FT3, free triiodothyronine; FT4, free thyroxine.

### 3.4 Pregnancy-loss ascertainment and RPL classification

A qualifying pregnancy loss was defined as a pregnancy loss occurring before 24 completed gestational weeks. A total of 264 qualifying pregnancy-loss episodes were documented, of which 208 (78.8%) were confirmed by ultrasonography and 56 (21.2%) were documented solely by serial serum human chorionic gonadotropin measurements and classified as biochemical pregnancy losses. Biochemical pregnancy losses were included in the primary RPL definition. In eight women, representing 11.4% of the RPL group, RPL classification depended on the inclusion of at least one biochemical pregnancy loss.

Among the 70 women with RPL, 35 (50.0%) had two qualifying pregnancy losses and 35 (50.0%) had three or more. Forty-six women (65.7%) had consecutive RPL, whereas 24 (34.3%) had non-consecutive RPL. Primary RPL was identified in 56 women (80.0%), and secondary RPL was identified in 14 women (20.0%); all women classified as having secondary RPL had experienced a live birth before fulfilling the RPL criteria.

At least one ART-associated pregnancy loss was documented in 18 women in the RPL group and 10 women in the non-RPL group (25.7% vs. 6.1%, P < 0.001). Among the 152 women with at least one qualifying pregnancy loss, the median gestational age at the most recent loss was 8.1 weeks in the RPL group and 7.6 weeks in the non-RPL group (P = 0.112). No pregnancy-loss episodes were unclassifiable on the basis of the available records (**Supplementary Table 1**).

### 3.5 Completeness of screening for recognized causes of RPL

All included participants underwent transvaginal ultrasonography, serum TSH measurement, prolactin testing, and evaluation for uncontrolled diabetes mellitus. Additional investigations were performed according to the available clinical records and clinical indications. Three-dimensional ultrasonography was documented in 51.4% of the RPL group and 36.4% of the non-RPL group (P = 0.042), whereas pelvic MRI was documented in 10.0% and 3.0%, respectively (P = 0.046).

Maternal karyotyping was available for 87.1% of women in the RPL group and 77.0% of those in the non-RPL group, and partner karyotyping was available for 84.3% and 74.5%, respectively. Assessment rates for lupus anticoagulant, anticardiolipin antibodies, anti-β2-glycoprotein I antibodies, TPOAb, TgAb, autoimmune disease, and inherited or acquired thrombophilia did not differ significantly between the groups. Before final enrollment, four patients were excluded because of uterine anatomical abnormalities, two because of chromosomal abnormalities in either partner, and four because of autoimmune disorders, thyroid dysfunction, or other conditions considered relevant to pregnancy outcomes. Undocumented assessments were not classified as normal or negative findings (**Supplementary Table 2**).

### 3.6 Timing of D-dimer measurement and data completeness

D-dimer measurements were available for all 235 participants. At the time of measurement, 210 women (89.4%) were nonpregnant, 15 (6.4%) were pregnant, and pregnancy status was undocumented for 10 (4.3%). Among women with a documented qualifying pregnancy loss, the median interval from the most recent loss to D-dimer measurement was 128 days overall and was shorter in the RPL group than in the non-RPL group [112 (IQR, 63–198) vs. 154 (IQR, 86–260) days, P = 0.041].

Measurements obtained during acute pregnancy loss, after uterine evacuation or surgery, during ovarian stimulation, shortly after oocyte retrieval or embryo transfer, or during anticoagulant or antiplatelet treatment were uncommon. A total of 196 women (83.4%) met all defined stable-baseline D-dimer criteria, including nonpregnant status and absence of acute pregnancy loss, recent reproductive procedures, active infection or inflammation, recent surgery, and current anticoagulant therapy. The proportion meeting these criteria did not differ significantly between the RPL and non-RPL groups (78.6% vs. 85.5%, P = 0.249) (**Supplementary Table 3**).

Complete data were available for the RPL outcome and all seven variables included in the final multivariable model; therefore, no imputation was performed for the primary analysis. FT4 was available for 227 women and was analyzed using available cases. Duration of infertility was analyzed only among the 125 women with a documented history of infertility and was not considered missing among women without infertility (**Supplementary Table 4**).

### 3.7 Univariable logistic regression analysis

In separate univariable logistic regression models, older age, higher BMI, nulliparity, a history of infertility, ART use, higher gravidity, longer duration of infertility, and greater ovarian volume were associated with higher odds of RPL status. A history of live birth was associated with lower odds of RPL status (OR = 0.26, 95% CI: 0.13–0.50, P < 0.001).

Among endocrine and metabolic variables, higher LH, LH/FSH ratio, total testosterone, fasting plasma glucose, fasting insulin, HOMA-IR, total cholesterol, triglycerides, and LDL-C were associated with higher odds of RPL status, whereas higher HDL-C was associated with lower odds. Fibrinogen and D-dimer were also associated with RPL status. The unadjusted OR for D-dimer was 18.42 per 1-mg/L increase (95% CI: 4.32–78.62, P < 0.001). Height, marital status, smoking, alcohol consumption, menstrual irregularity, antral follicle count, polycystic ovarian morphology, FSH, estradiol, prolactin, thyroid function parameters, prothrombin time, and activated partial thromboplastin time were not statistically associated with RPL status (**Table 3**).

**Table 3:** Univariable logistic regression analysis of factors associated with RPL.

Variable	Unit or reference category	No. analyzed	OR	95% CI	P value
Age, years	Per 1-year increase	235	1.09	1.03–1.15	0.004
Body weight, kg	Per 1-kg increase	235	1.03	1.00–1.06	0.023
Height, cm	Per 1-cm increase	235	0.98	0.94–1.03	0.388
BMI, kg/m²	Per 1-kg/m² increase	235	1.14	1.05–1.24	0.002
Married	Yes vs. no	235	1.35	0.65–2.78	0.417
Nulliparity	Yes vs. no	235	1.77	1.00–3.11	0.049
Smoking history	Yes vs. no	235	1.95	0.81–4.68	0.136
Alcohol consumption history	Yes vs. no	235	1.65	0.64–4.22	0.300
History of infertility	Yes vs. no	235	2.09	1.17–3.73	0.013
ART use	Yes vs. no	235	2.37	1.17–4.80	0.016
Menstrual irregularity	Oligomenorrhea or amenorrhea vs. regular	235	2.09	0.91–4.76	0.081
Gravidity	Per additional pregnancy	235	2.15	1.56–2.96	<0.001
History of live birth	Yes vs. no	235	0.26	0.13–0.50	<0.001
Duration of infertility, years	Per 1-year increase	125	1.36	1.10–1.68	0.005
Antral follicle count, n	Per additional follicle	235	1.04	0.99–1.09	0.076
Ovarian volume, mL	Per 1-mL increase	235	1.15	1.04–1.28	0.007
Polycystic ovarian morphology	Yes vs. no	235	1.65	0.71–3.82	0.240
LH, IU/L	Per 1-IU/L increase	235	1.10	1.03–1.18	0.006
FSH, IU/L	Per 1-IU/L increase	235	0.91	0.78–1.07	0.245
LH/FSH ratio	Per 1-unit increase	235	2.18	1.43–3.31	<0.001
Total testosterone, nmol/L	Per 1-nmol/L increase	235	1.93	1.25–2.98	0.003
Estradiol, pmol/L	Per 1-pmol/L increase	235	1.00	0.99–1.00	0.229
Prolactin, ng/mL	Per 1-ng/mL increase	235	1.03	0.98–1.08	0.191
Fasting plasma glucose, mmol/L	Per 1-mmol/L increase	235	1.82	1.16–2.85	0.009
Fasting insulin, μU/mL	Per 1-μU/mL increase	235	1.11	1.05–1.17	<0.001
HOMA-IR	Per 1-unit increase	235	1.60	1.28–2.00	<0.001
Total cholesterol, mmol/L	Per 1-mmol/L increase	235	1.39	1.00–1.94	0.048
Triglycerides, mmol/L	Per 1-mmol/L increase	235	2.43	1.53–3.85	<0.001
HDL-C, mmol/L	Per 1-mmol/L increase	235	0.18	0.06–0.59	0.005
LDL-C, mmol/L	Per 1-mmol/L increase	235	1.50	1.02–2.20	0.040
TSH, mIU/L	Per 1-mIU/L increase	235	1.32	0.99–1.76	0.059
FT3, pmol/L	Per 1-pmol/L increase	235	0.77	0.44–1.34	0.392
FT4, pmol/L	Per 1-pmol/L increase	227	0.93	0.81–1.07	0.308
Prothrombin time, s	Per 1-s increase	235	1.33	0.99–1.79	0.057
Activated partial thromboplastin time, s	Per 1-s increase	235	0.96	0.88–1.04	0.362
Fibrinogen, g/L	Per 1-g/L increase	235	1.86	1.20–2.88	0.005
D-dimer, mg/L	Per 1-mg/L increase	235	18.42	4.32–78.62	<0.001

Separate univariable logistic regression models were fitted with RPL status as the dependent variable and the non-RPL group as the reference outcome category. Oligomenorrhea and amenorrhea/marked irregularity were combined as menstrual irregularity. Duration of infertility was analyzed only among 125 women with a documented history of infertility. FT4 was analyzed using available data from 227 women.

RPL, recurrent pregnancy loss; OR, odds ratio; CI, confidence interval; BMI, body mass index; ART, assisted reproductive technology; LH, luteinizing hormone; FSH, follicle-stimulating hormone; HOMA-IR, homeostasis model assessment of insulin resistance; HDL-C, high-density lipoprotein cholesterol; LDL-C, low-density lipoprotein cholesterol; TSH, thyroid-stimulating hormone; FT3, free triiodothyronine; FT4, free thyroxine.

### 3.8 Multivariable logistic regression analysis and multicollinearity assessment

In the multivariable logistic regression model, age (adjusted OR = 1.06 per year, 95% CI: 1.01–1.12, P = 0.017), BMI (adjusted OR = 1.10 per kg/m², 95% CI: 1.02–1.19, P = 0.012), a history of infertility (adjusted OR = 2.01, 95% CI: 1.11–3.64, P = 0.021), LH/FSH ratio (adjusted OR = 1.77 per unit, 95% CI: 1.18–2.67, P = 0.006), HOMA-IR (adjusted OR = 1.41 per unit, 95% CI: 1.15–1.73, P = 0.001), and D-dimer (adjusted OR = 6.06 per 1-mg/L increase, 95% CI: 1.92–19.15, P = 0.002) remained independently associated with higher odds of RPL status. A history of live birth remained associated with lower odds of RPL status (adjusted OR = 0.35, 95% CI: 0.18–0.68, P = 0.002) (**Table 4**).

**Table 4:** Multivariable logistic regression analysis of factors independently associated with RPL.

Predictor	Unit or reference category	β	SE	Adjusted OR	95% CI	P value
Age, years	Per 1-year increase	0.062	0.026	1.06	1.01–1.12	0.017
BMI, kg/m²	Per 1-kg/m² increase	0.098	0.039	1.10	1.02–1.19	0.012
History of infertility	Yes vs. no	0.698	0.301	2.01	1.11–3.64	0.021
History of live birth	Yes vs. no	−1.057	0.349	0.35	0.18–0.68	0.002
LH/FSH ratio	Per 1-unit increase	0.573	0.207	1.77	1.18–2.67	0.006
HOMA-IR	Per 1-unit increase	0.341	0.103	1.41	1.15–1.73	0.001
D-dimer, mg/L	Per 1-mg/L increase	1.801	0.586	6.06	1.92–19.15	0.002

No substantial multicollinearity was identified among the seven variables in the final model. Tolerance values ranged from 0.658 to 0.877, and variance inflation factors ranged from 1.14 to 1.52 (**Supplementary Table 5**).

### 3.9 Development and apparent performance of the discriminative model

The final multivariable model incorporated age, BMI, history of infertility, history of live birth, LH/FSH ratio, HOMA-IR, and D-dimer. The model equation was: Logit(P) = −8.214 + 0.062 × age + 0.098 × BMI + 0.698 × history of infertility − 1.057 × history of live birth + 0.573 × LH/FSH ratio + 0.341 × HOMA-IR + 1.801 × D-dimer, where history of infertility and history of live birth were coded as 1 for yes and 0 for no, and P represented the estimated probability of being classified as having RPL in the study cohort.

**Figure 1:**
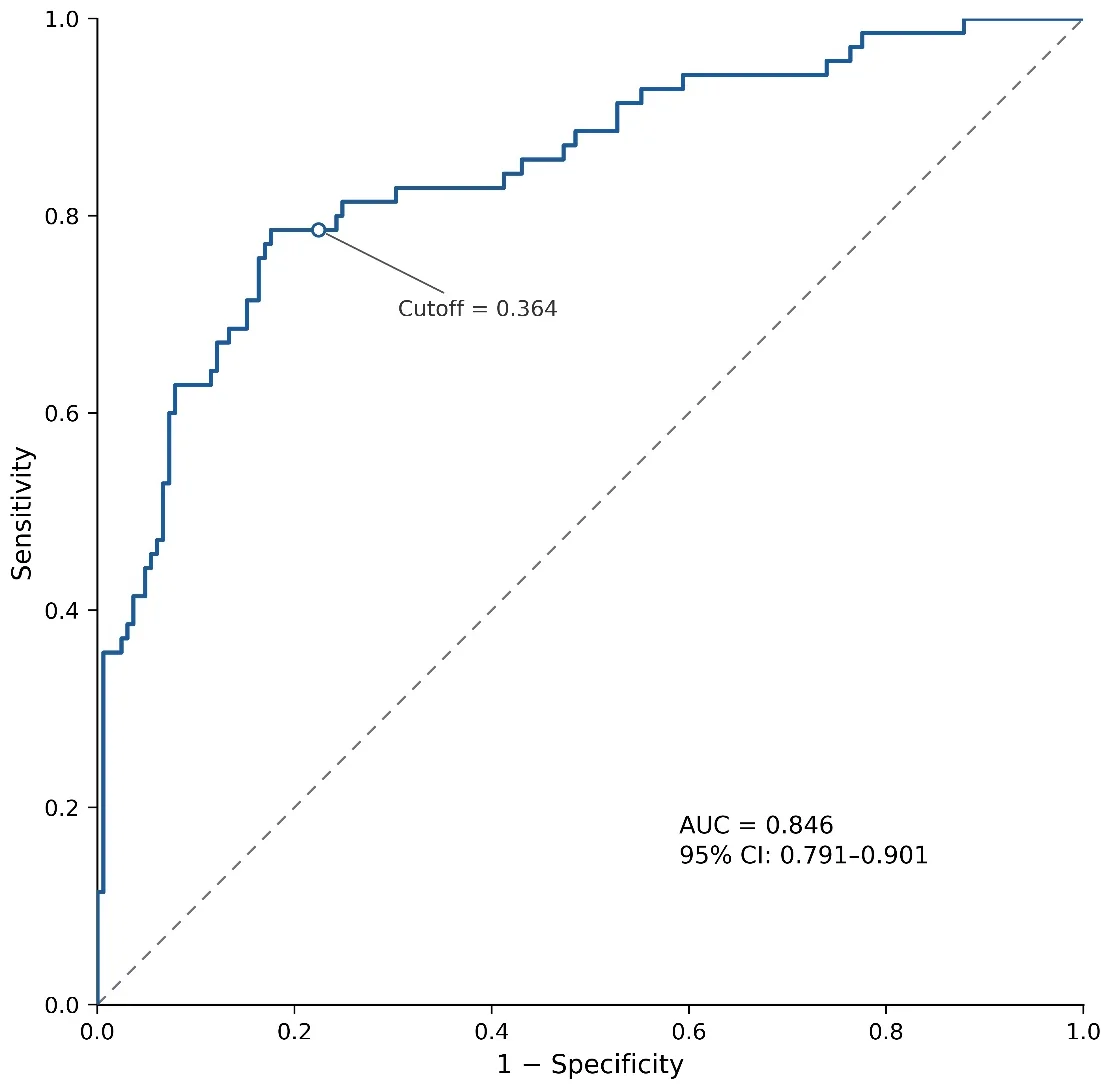
Receiver operating characteristic curve of the multivariable model for discriminating recurrent pregnancy loss status in women with polyendocrine metabolic ovarian syndrome.

The model showed an apparent AUC of 0.846 (95% CI, 0.791–0.901). At the predicted-probability cutoff of 0.364, the apparent sensitivity was 78.6%, specificity was 77.6%, positive predictive value was 59.8%, negative predictive value was 89.5%, and overall accuracy was 77.9% (**Figure 1**).

### 3.10 Sensitivity analyses

After biochemical pregnancy losses were excluded from the qualifying pregnancy-loss count, 62 women remained classified in the RPL group and 173 in the non-RPL group. In this reclassified analysis, the adjusted association for D-dimer was similar to that in the primary analysis (adjusted OR = 5.72, 95% CI: 1.75–18.69, P = 0.004), and the model AUC was 0.839.

When the analysis was restricted to the 196 women whose D-dimer measurements met all stable-baseline criteria, the adjusted OR for D-dimer was 5.49 (95% CI: 1.58–19.10, P = 0.007), and the AUC was 0.842. In the model excluding history of live birth, the adjusted OR for D-dimer was 5.83 (95% CI: 1.88–18.10, P = 0.002), with an AUC of 0.833. Exclusion of D-dimer from the model reduced the AUC to 0.824. Among the 174 women with complete documented screening for recognized causes of RPL, the adjusted OR for D-dimer was 6.31 (95% CI: 1.71–23.28, P = 0.006), and the AUC was 0.850. The direction of the associations and overall model discrimination were broadly similar across these analyses (**Supplementary Table 6**).

### 3.11 Bootstrap-corrected model performance

After bootstrap correction, the estimated AUC was 0.831 (95% CI: 0.770–0.885), corresponding to an estimated optimism of 0.015 relative to the apparent AUC. The bootstrap-corrected calibration intercept was 0.021 (95% CI: −0.140 to 0.176), the calibration slope was 0.887 (95% CI: 0.708–1.073), and the Brier score was 0.145 (95% CI: 0.122–0.169).

At the fixed probability cutoff of 0.364, the bootstrap-corrected sensitivity was 75.7% (95% CI: 64.5%–84.2%), specificity was 74.5% (95% CI: 67.3%–80.5%), positive predictive value was 55.7% (95% CI: 45.7%–65.2%), negative predictive value was 87.8% (95% CI: 81.5%–92.2%), and overall accuracy was 74.9% (95% CI: 68.8%–80.2%) (**Supplementary Table 7**).

## 4 Discussion

This study provides a multidimensional characterization of RPL status among women with PMOS by jointly evaluating reproductive history, endocrine and metabolic characteristics, and coagulation-related markers. Its principal novelty lies in incorporating D-dimer alongside routinely available variables reflecting adiposity, infertility history, gonadotropic balance, and insulin resistance, while defining the outcome as retrospectively documented RPL status rather than subsequent pregnancy loss (25, 26). The seven-variable model demonstrated good apparent discrimination, retained similar performance after bootstrap correction, and showed consistent findings across analyses addressing biochemical pregnancy losses, D-dimer measurement context, live-birth history, and etiological-screening completeness. Clinically, these findings emphasize that RPL status in women with PMOS is associated with a combination of reproductive, metabolic, endocrine, and coagulation-related features rather than a single abnormality. A structured assessment of these domains may therefore provide a more informative clinical profile of women presenting with a history of pregnancy loss. The model should be viewed as an internally validated means of distinguishing documented RPL status in comparable populations, providing a basis for standardized assessment and future prospective investigation rather than prediction of subsequent pregnancy outcomes.

The reproductive findings suggest that documented RPL status in women with PMOS is embedded within a distinct pattern of prior reproductive experience. Women in the RPL group were older and more frequently had infertility, previous assisted reproductive technology use, nulliparity, and a longer duration of infertility, whereas previous live birth was substantially less frequent. After adjustment, age and infertility history remained positively associated with RPL status, while previous live birth remained inversely associated. The association with age is consistent with the progressive decline in oocyte competence and the increasing probability of embryonic chromosomal abnormalities across the reproductive lifespan, although the present analysis cannot determine the mechanism underlying this relationship (27). Infertility history may identify women with more persistent reproductive dysfunction, but it should not be interpreted as an independent cause of repeated loss. Similarly, the inverse association with previous live birth requires careful interpretation because reproductive history is closely related to the distinction between primary and secondary RPL; notably, 80% of women with RPL in this cohort had primary RPL (28, 29). The univariable associations of gravidity, nulliparity, assisted reproductive technology use, and infertility duration may therefore partly reflect differences in reproductive exposure and clinical complexity rather than separate biological pathways. The endocrine and metabolic results further indicate that the PMOS phenotype associated with RPL status was characterized more strongly by functional hormonal and metabolic abnormalities than by ovarian morphology alone. Higher luteinizing hormone levels, luteinizing hormone/follicle-stimulating hormone ratios, testosterone concentrations, glycemic indices, and an adverse lipid profile were observed in women with RPL. However, only the luteinizing hormone/follicle-stimulating hormone ratio and homeostasis model assessment of insulin resistance remained associated after multivariable adjustment. This pattern suggests that gonadotropic imbalance and insulin resistance may summarize clinically relevant dimensions of PMOS heterogeneity more effectively than isolated hormone or lipid measurements. In contrast, antral follicle count, polycystic ovarian morphology, follicle-stimulating hormone, estradiol, prolactin, and thyroid-function indices were not associated with RPL status, indicating that the presence or morphological severity of polycystic ovaries does not necessarily parallel the burden of previous pregnancy loss (30, 31).

Among the coagulation-related variables, D-dimer showed the clearest association with documented RPL status. Women with RPL had higher mean D-dimer concentrations than those without RPL, whereas prothrombin time and activated partial thromboplastin time did not differ significantly. Fibrinogen was also higher in the RPL group in the unadjusted analysis, but D-dimer was the coagulation-related marker retained in the multivariable model. This pattern may indicate that variation in fibrin formation and degradation is more closely related to RPL status than conventional global clotting times; however, it does not establish hypercoagulability, placental microthrombosis, or any specific causal pathway (32, 33). D-dimer is a nonspecific marker influenced by pregnancy, recent pregnancy loss, surgery, inflammation, ovarian stimulation, reproductive procedures, and antithrombotic treatment (34, 35). Accordingly, its clinical context is essential when interpreting an isolated measurement. Several findings support the internal consistency of the observed association. Most measurements were obtained in nonpregnant women, and 83.4% of participants met the defined stable-baseline criteria. When the analysis was restricted to these participants, the adjusted association remained similar to that in the full cohort. The association also persisted after exclusion of biochemical pregnancy losses and among women with complete documented evaluation for recognized causes of RPL. Moreover, removing D-dimer from the seven-variable model reduced the area under the curve from 0.846 to 0.824, suggesting that D-dimer contributed some discriminatory information beyond the reproductive, endocrine, and metabolic variables included in the model. Nevertheless, this modest difference should not be interpreted as proof of incremental clinical utility, and the present findings do not support the use of D-dimer as a stand-alone diagnostic marker or as an indication for anticoagulant treatment. Rather, D-dimer should be regarded as an exploratory circulating marker associated with retrospectively documented RPL status whose biological and clinical significance requires further prospective evaluation.

Insulin resistance is a central metabolic feature of PMOS and may be accompanied by compensatory hyperinsulinemia. In the present study, higher homeostasis model assessment of insulin resistance was independently associated with documented RPL status, supporting the clinical relevance of metabolic assessment in this population (36). Lifestyle intervention remains fundamental to PMOS management, and insulin-sensitizing treatment may be considered in appropriately selected women with metabolic risk. Given the established susceptibility of women with PMOS to gestational dysglycemia, metabolic status should also be assessed before and during pregnancy. However, our retrospective findings do not demonstrate that reducing insulin resistance would prevent subsequent pregnancy loss, and prospective studies are needed to determine whether metabolic improvement translates into better reproductive outcomes. More broadly, circulating biomarkers have been investigated for the assessment of adverse pregnancy outcomes. Ding et al. (37) reported an elevated soluble fms-like tyrosine kinase-1/placental growth factor ratio in preeclampsia, illustrating the potential clinical relevance of measurable circulating biomarkers in pregnancy-related disorders. Nevertheless, this finding is not directly comparable with our results because preeclampsia and PMOS -associated RPL are distinct outcomes and the biomarkers reflect different biological processes. Unlike the sFlt-1/PlGF ratio, which reflects angiogenic and placental dysfunction, D-dimer is a nonspecific marker of fibrin turnover that is strongly influenced by the clinical context of measurement. Accordingly, its association with RPL status should be regarded as exploratory and should not be interpreted as evidence of prospective prediction or causality.

Several limitations should be acknowledged. First, the retrospective, single-center design may have introduced selection and information bias, residual confounding, and limited generalizability. RPL status was reconstructed from clinical records, and the cohort included heterogeneous RPL phenotypes. Although exclusion of biochemical pregnancy losses yielded similar findings, some outcome misclassification remains possible. Second, evaluation for recognized causes of RPL was not performed uniformly, so unrecognized alternative causes cannot be fully excluded. Third, D-dimer was not measured at standardized reproductive time points. Despite consistent results in the stable-baseline subgroup, residual effects of pregnancy status, time since pregnancy loss, reproductive procedures, inflammation, surgery, or antithrombotic therapy may remain. D-dimer should therefore be regarded as a context-dependent marker associated with documented RPL status rather than as a causal or prognostic factor. Fourth, the sample size was modest, and potentially relevant variables, including inflammatory markers, detailed androgen profiles, lifestyle factors, and PMOS phenotypes, were unavailable. Finally, although sensitivity analyses and bootstrap validation supported internal robustness, the model has not been externally validated and should not be interpreted as predicting future pregnancy loss. Larger prospective multicenter studies using standardized assessments and predefined measurement time points are needed to externally validate the model, evaluate its clinical utility, and determine whether the identified factors are associated with subsequent pregnancy outcomes.

## 5 Conclusion

In conclusion, RPL status among women with PMOS was associated with older age, higher BMI, a history of infertility, a higher LH/FSH ratio, greater insulin resistance, and higher D-dimer levels, whereas a history of live birth was inversely associated with RPL status. The resulting seven-variable model showed good apparent and bootstrap-corrected discrimination for distinguishing women with and without a documented history of RPL in this cohort. Given the retrospective, single-center design, these findings reflect associations rather than causal or prospective predictive relationships. Prospective multicenter studies are needed to externally validate the model, evaluate its prediction of subsequent pregnancy outcomes, and clarify the clinical relevance of modifiable factors.

## Data Availability

The raw data supporting the conclusions of this article will be made available by the authors, without undue reservation.
