# Supplementary material for: Clinical factors associated with recurrent pregnancy loss status in women with polyendocrine metabolic ovarian syndrome
Source: Front Endocrinol (Lausanne). 2026 Sep 10;17:1897833. doi: 10.3389/fendo.2026.1897833 (PMC13600818; doi:10.3389/fendo.2026.1897833)
Supplement: Supplementary file 1 [file Table1.docx]

Supplementary Table S1. Pregnancy-loss ascertainment and RPL classification

| Characteristic | Overall cohort (n = 235) | RPL group (n = 70) | Non-RPL group (n = 165) | P value |
| --- | --- | --- | --- | --- |
| Gestational-age threshold for a qualifying pregnancy loss | <24 completed weeks | <24 completed weeks | <24 completed weeks | — |
| Total documented pregnancy-loss episodes, n | 264 | 182 | 82 | — |
| Ultrasound-confirmed pregnancy-loss episodes, n/N (%) | 208/264 (78.8) | 143/182 (78.6) | 65/82 (79.3) | — |
| hCG-only pregnancy-loss episodes, n/N (%) | 56/264 (21.2) | 39/182 (21.4) | 17/82 (20.7) | — |
| Biochemical pregnancy-loss episodes counted toward the primary RPL definition, n | 56 | 39 | 17 | — |
| Biochemical pregnancy-loss episodes not counted toward the primary RPL definition, n | 0 | 0 | 0 | — |
| RPL classification dependent on ≥1 biochemical pregnancy loss, n (%) | 8 (3.4) | 8 (11.4) | 0 (0.0) | <0.001 |
| Number of qualifying pregnancy losses, n (%) |  |  |  | — |
| └ 0 | 83 (35.3) | 0 (0.0) | 83 (50.3) |  |
| └ 1 | 82 (34.9) | 0 (0.0) | 82 (49.7) |  |
| └ 2 | 35 (14.9) | 35 (50.0) | 0 (0.0) |  |
| └ ≥3 | 35 (14.9) | 35 (50.0) | 0 (0.0) |  |
| Consecutive RPL, n (%) | 46 (19.6) | 46 (65.7) | Not applicable | — |
| Non-consecutive RPL, n (%) | 24 (10.2) | 24 (34.3) | Not applicable | — |
| Primary RPL, n (%) | 56 (23.8) | 56 (80.0) | Not applicable | — |
| Secondary RPL, n (%) | 14 (6.0) | 14 (20.0) | Not applicable | — |
| Live birth before RPL, n (%) | 14 (6.0) | 14 (20.0) | Not applicable | — |
| At least one ART-associated pregnancy loss, n (%) | 28 (11.9) | 18 (25.7) | 10 (6.1) | <0.001 |
| Gestational age at the most recent qualifying pregnancy loss, weeks, median (IQR) | 7.9 (6.0–10.0), n = 152 | 8.1 (6.1–10.2), n = 70 | 7.6 (5.9–9.6), n = 82 | 0.112 |
| Unclassifiable pregnancy-loss episodes, n (%) | 0 (0.0) | 0 (0.0) | 0 (0.0) | — |

Notes: Episode-level variables use the number of documented pregnancy-loss episodes as the denominator; all other variables are reported at the patient level. Consecutive RPL was defined as at least two qualifying pregnancy losses occurring in successive pregnancies without an intervening ongoing pregnancy or live birth. Non-consecutive RPL was defined as at least two qualifying losses separated by one or more pregnancies not ending in qualifying loss. Primary RPL referred to RPL occurring in women with no previous live birth; secondary RPL referred to RPL occurring after at least one previous live birth. Pregnancy losses documented solely by serial serum hCG measurements without ultrasonographic confirmation were classified as biochemical pregnancy losses.

Supplementary Table S2. Completeness of screening for recognized causes of RPL among included participants

Panel A. Screening completed among included participants

| Condition evaluated | Examination or diagnostic method | RPL group assessed, n/N (%) | RPL group not assessed, n/N (%) | Non-RPL group assessed, n/N (%) | Non-RPL group not assessed, n/N (%) | P value for assessment rate |
| --- | --- | --- | --- | --- | --- | --- |
| Uterine anatomical abnormalities | Transvaginal ultrasonography | 70/70 (100.0) | 0/70 (0.0) | 165/165 (100.0) | 0/165 (0.0) | — |
| Congenital uterine anomalies | Three-dimensional ultrasonography | 36/70 (51.4) | 34/70 (48.6) | 60/165 (36.4) | 105/165 (63.6) | 0.042 |
| Intrauterine cavity abnormalities | Hysteroscopy | 16/70 (22.9) | 54/70 (77.1) | 22/165 (13.3) | 143/165 (86.7) | 0.082 |
| Uterine cavity and tubal abnormalities | Hysterosalpingography | 10/70 (14.3) | 60/70 (85.7) | 18/165 (10.9) | 147/165 (89.1) | 0.511 |
| Complex pelvic anatomical abnormalities | Pelvic MRI | 7/70 (10.0) | 63/70 (90.0) | 5/165 (3.0) | 160/165 (97.0) | 0.046 |
| Maternal chromosomal abnormalities | Peripheral blood karyotyping | 61/70 (87.1) | 9/70 (12.9) | 127/165 (77.0) | 38/165 (23.0) | 0.078 |
| Partner chromosomal abnormalities | Peripheral blood karyotyping | 59/70 (84.3) | 11/70 (15.7) | 123/165 (74.5) | 42/165 (25.5) | 0.125 |
| Antiphospholipid syndrome | Lupus anticoagulant | 66/70 (94.3) | 4/70 (5.7) | 144/165 (87.3) | 21/165 (12.7) | 0.164 |
| Antiphospholipid syndrome | Anticardiolipin antibody IgG/IgM | 65/70 (92.9) | 5/70 (7.1) | 140/165 (84.8) | 25/165 (15.2) | 0.133 |
| Antiphospholipid syndrome | Anti-β2-glycoprotein I antibody IgG/IgM | 63/70 (90.0) | 7/70 (10.0) | 135/165 (81.8) | 30/165 (18.2) | 0.169 |
| Thyroid dysfunction | Serum TSH | 70/70 (100.0) | 0/70 (0.0) | 165/165 (100.0) | 0/165 (0.0) | — |
| Thyroid dysfunction | Serum FT4 | 68/70 (97.1) | 2/70 (2.9) | 159/165 (96.4) | 6/165 (3.6) | 1.000 |
| Thyroid autoimmunity | TPOAb | 61/70 (87.1) | 9/70 (12.9) | 130/165 (78.8) | 35/165 (21.2) | 0.148 |
| Thyroid autoimmunity | TgAb | 59/70 (84.3) | 11/70 (15.7) | 126/165 (76.4) | 39/165 (23.6) | 0.223 |
| Hyperprolactinemia | Serum prolactin | 70/70 (100.0) | 0/70 (0.0) | 165/165 (100.0) | 0/165 (0.0) | — |
| Uncontrolled diabetes mellitus | Fasting glucose, HbA1c, and medical records | 70/70 (100.0) | 0/70 (0.0) | 165/165 (100.0) | 0/165 (0.0) | — |
| Autoimmune disease | Medical history and clinically indicated antibody testing | 64/70 (91.4) | 6/70 (8.6) | 146/165 (88.5) | 19/165 (11.5) | 0.645 |
| Inherited or acquired thrombophilia | Institutional thrombophilia panel | 60/70 (85.7) | 10/70 (14.3) | 127/165 (77.0) | 38/165 (23.0) | 0.158 |

Panel B. Recognized conditions leading to exclusion before final enrollment

| Exclusion category | Patients excluded, n |
| --- | --- |
| Uterine anatomical abnormalities | 4 |
| Chromosomal abnormality in either partner | 2 |
| Overt thyroid dysfunction | 2 |
| Isolated thyroid autoantibody positivity with normal thyroid function | 1 |
| Other autoimmune or clinically relevant conditions | 1 |
| Total etiological exclusions | 10 |

Notes: P values in Panel A compare assessment rates between the RPL and non-RPL groups and were calculated using Pearson’s chi-square test or Fisher’s exact test, as appropriate. Participants with documented findings meeting the exclusion criteria were excluded before final group assignment. Accordingly, among included participants who underwent a given etiological assessment, no corresponding exclusionary condition was identified. An undocumented assessment was classified as not assessed and was not interpreted as a normal or negative result.

Supplementary Table S3. Timing and clinical context of D-dimer measurements

| Measurement characteristic | Overall cohort (n = 235) | RPL group (n = 70) | Non-RPL group (n = 165) | P value |
| --- | --- | --- | --- | --- |
| D-dimer available, n (%) | 235 (100.0) | 70 (100.0) | 165 (100.0) | — |
| Pregnancy status at measurement, n (%) |  |  |  | 0.334 |
| └ Nonpregnant | 210 (89.4) | 60 (85.7) | 150 (90.9) |  |
| └ Pregnant | 15 (6.4) | 7 (10.0) | 8 (4.8) |  |
| └ Undocumented | 10 (4.3) | 3 (4.3) | 7 (4.2) |  |
| Days from the most recent qualifying pregnancy loss, median (IQR) | 128 (74–231), n = 152 | 112 (63–198), n = 70 | 154 (86–260), n = 82 | 0.041 |
| Measurement during the acute pregnancy-loss phase, n (%) | 9 (3.8) | 5 (7.1) | 4 (2.4) | 0.131 |
| Measurement within 30 days after uterine evacuation or surgery, n (%) | 10 (4.3) | 5 (7.1) | 5 (3.0) | 0.169 |
| Measurement during ovarian stimulation, n (%) | 8 (3.4) | 4 (5.7) | 4 (2.4) | 0.243 |
| Measurement within 7 days after oocyte retrieval, n (%) | 5 (2.1) | 3 (4.3) | 2 (1.2) | 0.158 |
| Measurement within 14 days after embryo transfer, n (%) | 7 (3.0) | 4 (5.7) | 3 (1.8) | 0.201 |
| Anticoagulant use at measurement, n (%) | 8 (3.4) | 4 (5.7) | 4 (2.4) | 0.243 |
| Antiplatelet use at measurement, n (%) | 6 (2.6) | 3 (4.3) | 3 (1.8) | 0.366 |
| Active infection or inflammatory condition, n (%) | 7 (3.0) | 3 (4.3) | 4 (2.4) | 0.428 |
| Non-obstetric surgery within 30 days before measurement, n (%) | 6 (2.6) | 3 (4.3) | 3 (1.8) | 0.366 |
| Met standardized stable-baseline criteria, n (%) | 196 (83.4) | 55 (78.6) | 141 (85.5) | 0.249 |
| Measurement context undocumented, n (%) | 10 (4.3) | 3 (4.3) | 7 (4.2) | 1.000 |

Supplementary Table S4. Data completeness and missing-data handling for variables used in descriptive and regression analyses

| Variable | Expected n | Available n | Missing in RPL group, n/N (%) | Missing in non-RPL group, n/N (%) | Total missing, n/N (%) | Statistical handling |
| --- | --- | --- | --- | --- | --- | --- |
| RPL classification | 235 | 235 | 0/70 (0.0) | 0/165 (0.0) | 0/235 (0.0) | Primary binary outcome |
| Age | 235 | 235 | 0/70 (0.0) | 0/165 (0.0) | 0/235 (0.0) | Included in the primary model |
| Body weight | 235 | 235 | 0/70 (0.0) | 0/165 (0.0) | 0/235 (0.0) | Descriptive and univariable analyses |
| Height | 235 | 235 | 0/70 (0.0) | 0/165 (0.0) | 0/235 (0.0) | Descriptive and univariable analyses |
| BMI | 235 | 235 | 0/70 (0.0) | 0/165 (0.0) | 0/235 (0.0) | Included in the primary model |
| Married | 235 | 235 | 0/70 (0.0) | 0/165 (0.0) | 0/235 (0.0) | Descriptive and univariable analyses |
| Nulliparity | 235 | 235 | 0/70 (0.0) | 0/165 (0.0) | 0/235 (0.0) | Descriptive and univariable analyses |
| Smoking history | 235 | 235 | 0/70 (0.0) | 0/165 (0.0) | 0/235 (0.0) | Descriptive and univariable analyses |
| Alcohol consumption history | 235 | 235 | 0/70 (0.0) | 0/165 (0.0) | 0/235 (0.0) | Descriptive and univariable analyses |
| History of infertility | 235 | 235 | 0/70 (0.0) | 0/165 (0.0) | 0/235 (0.0) | Included in the primary model |
| ART use | 235 | 235 | 0/70 (0.0) | 0/165 (0.0) | 0/235 (0.0) | Descriptive and univariable analyses |
| Menstrual pattern | 235 | 235 | 0/70 (0.0) | 0/165 (0.0) | 0/235 (0.0) | Descriptive and univariable analyses |
| Gravidity | 235 | 235 | 0/70 (0.0) | 0/165 (0.0) | 0/235 (0.0) | Descriptive and univariable analyses |
| Number of documented pregnancy losses | 235 | 235 | 0/70 (0.0) | 0/165 (0.0) | 0/235 (0.0) | Used to determine RPL classification |
| History of live birth | 235 | 235 | 0/70 (0.0) | 0/165 (0.0) | 0/235 (0.0) | Included in the primary model |
| Duration of infertility | 125 | 125 | 0/46 (0.0) | 0/79 (0.0) | 0/125 (0.0) | Restricted to women with infertility |
| Antral follicle count | 235 | 235 | 0/70 (0.0) | 0/165 (0.0) | 0/235 (0.0) | Descriptive and univariable analyses |
| Ovarian volume | 235 | 235 | 0/70 (0.0) | 0/165 (0.0) | 0/235 (0.0) | Descriptive and univariable analyses |
| Polycystic ovarian morphology | 235 | 235 | 0/70 (0.0) | 0/165 (0.0) | 0/235 (0.0) | Descriptive and univariable analyses |
| LH | 235 | 235 | 0/70 (0.0) | 0/165 (0.0) | 0/235 (0.0) | Descriptive and univariable analyses |
| FSH | 235 | 235 | 0/70 (0.0) | 0/165 (0.0) | 0/235 (0.0) | Descriptive and univariable analyses |
| LH/FSH ratio | 235 | 235 | 0/70 (0.0) | 0/165 (0.0) | 0/235 (0.0) | Included in the primary model |
| Total testosterone | 235 | 235 | 0/70 (0.0) | 0/165 (0.0) | 0/235 (0.0) | Descriptive and univariable analyses |
| Estradiol | 235 | 235 | 0/70 (0.0) | 0/165 (0.0) | 0/235 (0.0) | Descriptive and univariable analyses |
| Prolactin | 235 | 235 | 0/70 (0.0) | 0/165 (0.0) | 0/235 (0.0) | Descriptive and univariable analyses |
| Fasting plasma glucose | 235 | 235 | 0/70 (0.0) | 0/165 (0.0) | 0/235 (0.0) | Descriptive and univariable analyses |
| Fasting insulin | 235 | 235 | 0/70 (0.0) | 0/165 (0.0) | 0/235 (0.0) | Descriptive and univariable analyses |
| HOMA-IR | 235 | 235 | 0/70 (0.0) | 0/165 (0.0) | 0/235 (0.0) | Included in the primary model |
| Total cholesterol | 235 | 235 | 0/70 (0.0) | 0/165 (0.0) | 0/235 (0.0) | Descriptive and univariable analyses |
| Triglycerides | 235 | 235 | 0/70 (0.0) | 0/165 (0.0) | 0/235 (0.0) | Descriptive and univariable analyses |
| HDL-C | 235 | 235 | 0/70 (0.0) | 0/165 (0.0) | 0/235 (0.0) | Descriptive and univariable analyses |
| LDL-C | 235 | 235 | 0/70 (0.0) | 0/165 (0.0) | 0/235 (0.0) | Descriptive and univariable analyses |
| TSH | 235 | 235 | 0/70 (0.0) | 0/165 (0.0) | 0/235 (0.0) | Descriptive and univariable analyses |
| FT3 | 235 | 235 | 0/70 (0.0) | 0/165 (0.0) | 0/235 (0.0) | Descriptive and univariable analyses |
| FT4 | 235 | 227 | 2/70 (2.9) | 6/165 (3.6) | 8/235 (3.4) | Available-case analyses |
| Prothrombin time | 235 | 235 | 0/70 (0.0) | 0/165 (0.0) | 0/235 (0.0) | Descriptive and univariable analyses |
| Activated partial thromboplastin time | 235 | 235 | 0/70 (0.0) | 0/165 (0.0) | 0/235 (0.0) | Descriptive and univariable analyses |
| Fibrinogen | 235 | 235 | 0/70 (0.0) | 0/165 (0.0) | 0/235 (0.0) | Descriptive and univariable analyses |
| D-dimer | 235 | 235 | 0/70 (0.0) | 0/165 (0.0) | 0/235 (0.0) | Included in the primary model |

Notes: No imputation was performed for the primary model because complete data were available for all seven final predictors and the RPL outcome. Duration of infertility was applicable only to women with infertility and was not considered missing among women without infertility. Etiological screening completeness is reported separately in Supplementary Table S2.

Supplementary Table S5. Multicollinearity diagnostics for the final multivariable model

| Predictor | Coding or scale | Tolerance | Variance inflation factor |
| --- | --- | --- | --- |
| Age | Continuous; per 1-year increase | 0.781 | 1.28 |
| BMI | Continuous; per 1-kg/m² increase | 0.699 | 1.43 |
| History of infertility | Binary; yes = 1, no = 0 | 0.741 | 1.35 |
| History of live birth | Binary; yes = 1, no = 0 | 0.690 | 1.45 |
| LH/FSH ratio | Continuous; per 1-unit increase | 0.813 | 1.23 |
| HOMA-IR | Continuous; per 1-unit increase | 0.658 | 1.52 |
| D-dimer | Continuous; per 1-mg/L increase | 0.877 | 1.14 |

Supplementary Table S6. Sensitivity analyses

| Analysis | Population/model definition | No. analyzed | RPL group, n | Non-RPL group, n | Adjusted OR for D-dimer | 95% CI | P value | Model AUC | 95% CI for AUC |
| --- | --- | --- | --- | --- | --- | --- | --- | --- | --- |
| Primary analysis | Seven-variable model in the full cohort | 235 | 70 | 165 | 6.06 | 1.92–19.15 | 0.002 | 0.846 | 0.791–0.901 |
| Reclassification after excluding biochemical pregnancy losses | RPL status was reassigned after biochemical pregnancy losses were excluded from the qualifying pregnancy-loss count | 235 | 62 | 173 | 5.72 | 1.75–18.69 | 0.004 | 0.839 | 0.781–0.897 |
| Restricted to standardized nonpregnant D-dimer measurements | Restricted to participants who met all prespecified stable-baseline D-dimer criteria | 196 | 55 | 141 | 5.49 | 1.58–19.10 | 0.007 | 0.842 | 0.782–0.902 |
| Model excluding history of live birth | Six-variable model retaining all primary predictors except history of live birth | 235 | 70 | 165 | 5.83 | 1.88–18.10 | 0.002 | 0.833 | 0.775–0.891 |
| Model excluding D-dimer | Six-variable model retaining all primary predictors except D-dimer | 235 | 70 | 165 | — | — | — | 0.824 | 0.764–0.884 |
| Restricted to participants with complete documented RPL-cause screening | Required documented uterine assessment, maternal and partner karyotyping, antiphospholipid antibody testing, TSH, and FT4 | 174 | 53 | 121 | 6.31 | 1.71–23.28 | 0.006 | 0.850 | 0.786–0.914 |

Notes: Unless otherwise specified, all sensitivity models included age, BMI, history of infertility, history of live birth, LH/FSH ratio, HOMA-IR, and D-dimer. When a predictor was excluded, all remaining primary-model predictors were retained. AUCs were obtained from receiver operating characteristic curve analysis, and their 95% confidence intervals were calculated using the DeLong method. Stable-baseline D-dimer criteria comprised nonpregnant status; no acute pregnancy loss; no recent uterine evacuation, surgery, ovarian stimulation, oocyte retrieval, or embryo transfer; no active infection or inflammatory condition; and no current anticoagulant treatment.

Abbreviations: RPL, recurrent pregnancy loss; OR, odds ratio; CI, confidence interval; AUC, area under the receiver operating characteristic curve; BMI, body mass index; LH, luteinizing hormone; FSH, follicle-stimulating hormone; HOMA-IR, homeostasis model assessment of insulin resistance; TSH, thyroid-stimulating hormone; FT4, free thyroxine.

Supplementary Table S7. Apparent and bootstrap-corrected performance of the final model

| Performance measure | Apparent estimate (95% CI) | Estimated optimism | Bootstrap-corrected estimate (95% CI) | Performance measure |
| --- | --- | --- | --- | --- |
| AUC | 0.846 (0.791–0.901) | 0.015 | 0.831 (0.770–0.885) | AUC |
| Calibration intercept | 0 | −0.021 | 0.021 (−0.140 to 0.176) | Calibration intercept |
| Calibration slope | 1 | 0.113 | 0.887 (0.708–1.073) | Calibration slope |
| Brier score | 0.138 | −0.007 | 0.145 (0.122–0.169) | Brier score |
| Fixed predicted-probability cutoff | 0.364 | — | 0.364 | Fixed predicted-probability cutoff |
| True positives, n | 55 | — | — | True positives, n |
| False negatives, n | 15 | — | — | False negatives, n |
| True negatives, n | 128 | — | — | True negatives, n |
| False positives, n | 37 | — | — | False positives, n |
| Sensitivity at cutoff 0.364 | 78.60% | 2.9 percentage points | 75.7% (64.5%–84.2%) | Sensitivity at cutoff 0.364 |
| Specificity at cutoff 0.364 | 77.60% | 3.1 percentage points | 74.5% (67.3%–80.5%) | Specificity at cutoff 0.364 |
| Positive predictive value | 59.80% | 4.1 percentage points | 55.7% (45.7%–65.2%) | Positive predictive value |
| Negative predictive value | 89.50% | 1.7 percentage points | 87.8% (81.5%–92.2%) | Negative predictive value |
| Overall accuracy | 77.90% | 3.0 percentage points | 74.9% (68.8%–80.2%) | Overall accuracy |
| Youden index | 0.561 | 0.059 | 0.502 (0.344–0.630) | Youden index |
| Balanced accuracy | 0.781 | 0.03 | 0.751 (0.688–0.804) | Balanced accuracy |
